# LncRNA MAGI2-AS3 is downregulated in non-small cell lung cancer and may be a sponge of miR-25

**DOI:** 10.1186/s12890-020-1064-7

**Published:** 2020-03-05

**Authors:** Yutong Sui, Wencheng Chi, Li Feng, Jiakang Jiang

**Affiliations:** 1 National Cancer Center/National Clinical Research Center for Cancer/Cancer Hospital, Chinese Academy of Medical Sciences and Peking Union Medical College, Beijing, 100021 China; 2 Heilongjiang University of Chinese Medicine, Harbin, 150040 Heilongjiang province China; 3 First Affiliated Hospital, Heilongjiang University of Chinese Medicine, Heping Road 26, Harbin, 150040 Heilongjiang province China

**Keywords:** Non-small cell lung cancer, MAGI2-AS3, miR-25, RECK, Sponge

## Abstract

**Background:**

This study aimed to investigate the role of lncRNA MAGI2-AS3 in non-small cell lung cancer (NSCLC).

**Methods:**

Expression levels of MAGI2-AS3 and RECK mRNA in two types of tissues (non-tumor and NCSLC) were measured by qPCR. To further investigate the interaction between MAGI2-AS3 and RECK, MAGI2-AS3 and RECK expression vectors were transfected into H1993 cells.

**Results:**

We found that MAGI2-AS3 and RECK were upregulated and positively correlated in NSCLC. In NSCLC cells, MAGI2-AS3 overexpression led to upregulated RECK. Bioinformatics analysis showed that MAGI2-AS3 may bind miR-25, which can directly target RECK. In NSCLC cells, miR-25 overexpression led to downregulated RECK and attenuated the effects of MAGI2-AS3 overexpression, while MAGI2-AS3 and miR-25 failed to affect each other. Cell invasion and migration analysis showed decreased NSCLC cell invasion and migration rates after MAGI2-AS3 and RECK overexpression. MiR-25 showed opposite role and reduced the effects of MAGI2-AS3 overexpression.

**Conclusion:**

Therefore, MAGI2-AS3 may sponge miR-25 to upregulate RECK, thereby inhibiting NSCLC cell invasion and migration.

**Trial registration:**

HLJCM20163358592, registered by First Affiliated Hospital, Heilongjiang University of Chinese Medicine at March 3, 2016, prospectively.

## Background

Lung cancer is the most common cause of deaths in cancer patients [1]. Every year, lung cancer causes more than 1.6 million deaths, which accounts for about 20% of all deaths causes by all cancers [2]. Due to the fact that most lung cancer patients are diagnosed when tumor metastasis already exist, it is estimated that only less than 5% of lung cancer patients can live longer than 5 years after the initial diagnosis [3]. Non-small-cell lung cancers (NSCLCs) are the major subgroup of lung cancer [3]. Although smoking is the major risk factor for NSCLC [4], NSCLC also affects never-smokers [5], indicating the involvement of genetic factors in this disease [6].

RECK, also known as reversion-inducing-cysteine-rich protein with kazal motifs, has been characterized as a metastasis suppressor [7]. RECK interacts matrix metalloproteinase to facilitate cell invasion [8, 9]. RECK in cancer can be targeted by certain oncogenic miRNAs, such as miRNA-182-5p and miR-25 [10, 11]. In gastric cancer, miR-25 targets to RECK to promote cancer cell motility and growth [11]. It is known that the functions of miRNAs can be attenuated by their sponges, such as long (> 200 nt) non-coding RNAs [12]. LncRNA MAGI2-AS3 has been characterized as a oncogenic lncRNA in breast cancer, bladder cancer and liver cancer [13–15]. Our bioinformatics analysis showed that MAGI2-AS3 may interact with miR-25. This study aimed to investigate the interaction between miR-25 and MAGI2-AS3 in NSCLC.

## Methods

### Study subjects

The subjects of the present study were 78 NSCLC patients (gender: 48 males and 30 females; age: 34 to 66 years; mean: 50.2 ± 5.6 year) who were admitted to Heilongjiang University of Chinese Medicine between May 2016 and January 2019. Those 78 patients were selected according to: inclusion criteria: 1) newly diagnosed NSCLC; 2) no therapies received before admission, and exclusion criteria: 1) complicated with other diseases; 2) recurrent NSCLC; 3) therapies were initiated; 4) transferred from other hospitals. Those 78 NSCLC included 14, 15, 23 and 26 cases at stage (AJCC) I-IV, respectively. All the 78 patients were informed with experimental principle. Before the admission of patients, Ethics Committee of Heilongjiang University of Chinese Medicine approved this study.

### NSCLC tissues and cells

All in vitro studies were performed using H1993 human NSCLC (ATCC, USA) cell lines. Cells were cultivated in the mixture of 90% RPMI-1640 medium and 10% (W/V) FBS. Cells were cultivated at 37 °C in an incubator (95% humidity and 5% CO_2_).

To perform in vivo gene expression analysis, biopsy was performed on all patients to obtain both non-tumor and NSCLC tissues. Weight of tissues ranged from 16 mg to 21 mg. Tissues were tested by pathologists. Before used, all tissues were kept in liquid nitrogen.

### Prediction of the interaction between MAGI2-AS3 and miR-25

To predict the possible interaction between MAGI2-AS3 (NCBI Accession: NR_038343.2) and miR-25 (miRbase Accession: MI0000082), MAGI2-AS3 (long sequence) and miR-25 (short sequence) were inputted into IntaRNA (http://rna.informatik.uni-freiburg.de/IntaRNA/Input.jsp) RNA-RNA interaction online prediction program. All the parameters were default.

### Vectors and miRNA mimic

Negative control miRNA and miR-25 mimic (miRbase Accession: MI0000082) were from Sigma-Aldrich (USA). MAGI2-AS3 (NCBI Accession: NR_038343.2) and RECK (NCBI Accession: NM_021111.3) expression pcDNA3 vectors were constructed by GenePharma (Shanghai, China).

### Transient transfections

H1993 cells were harvested at confluence of 70–80%. Cells were counted and 5 × 10^5^ cells were transfected with 40 nM negative control miRNA (negative control, NC), or miR-25 mimic (miR-25 group), or 10 nM empty pcDNA3 vector (NC), or 10 nM MAGI2-AS3 (MAGI2-AS3 group) or RECK expression (RECK group) pcDNA3 vectors through lipofectamine 2000 (Sangon) mediated transient transfections. Cells without transfections were control (C) group cells. The interval between transfections and following experiments was 24 h.

### RNA extractions

RNAs in H1993 cells as well as non-tumor and NSCLC tissues were extracted using Trizol (Invitrogen, USA). All RNA samples were precipitated using 85% ethanol. 85% ethanol was also used in the washing step. With the use of 85% ethanol, all types of RNAs including miRNAs were harvested.

### qPCR

The total RNA samples were subjected to digestion with DNase I for 1 h at 37 °C. iScript cDNA Synthesis Kit (Bio-Rad, USA) and qScript microRNA cDNA Synthesis Kit (Quantabio, USA) were used to perform total RNA and miRNA reverse transcriptions, respectively. To measure the expression levels of MAGI2-AS3 and RECK mRNA, all qPCR reaction mixtures were prepared using BlazeTaq™ SYBR Green qPCR Mix (Genecopoeia, Guangzhou, China) with GAPDH as endogenous control. To measure the expression levels of miR-25, all qPCR reaction mixtures were prepared using miScript SYBR Green PCR Kit (QIAGEN) with U6 as endogenous control. All PCR reactions were performed 3 times. 2^-ΔΔCT^ method was used for data normalizations. The sample with the biggest ΔCT value was set to value “1”, and all other samples were normalized to this sample to calculate the relative expression levels. Amplification efficiencies of all primer pairs were between 95 and 100%. PCR products were randomly selected and sequenced. All sequenced PCR products were correct.

### Western blot

Total proteins in H1993 cells were extracted using RIPA solution (Sangon). Before, electrophoresis (10% SDS-PAGE gel), all protein samples were denatured in boiled water for 10 min. After electrophoresis, proteins were transferred to PVDF membranes, followed by blocking in 5% non-fat milk for 1 h. PVDF membranes were first blotted by rabbit primary antibodies of GAPDH (1: 1300, ab37168, Abcam) and RECK (1: 1300, ab115844, Abcam) for at least 15 h at 4 °C, followed by blotting with HRP goat anti-rabbit (IgG) antibody (1:1000; ab6721; Abcam) for 90 min at 25 °C. Signals were developed using RapidStep™ ECL detection reagent from EMD Millipore. Image J v1.46 software was used to normalize gray values. It is worth noting that with β-actin as internal control, the same data were obtained.

### Cell invasion and migration analysis

H1993 cells were mixed with serum-free RPMI-1640 medium to prepare single cell suspensions (3 × 10^4^ cells per well). 0.1 ml suspension (serum-free) was added into Transwell upper chamber. The low chamber was added with RPMI-1640 medium containing 20% (W/V) FBS. Upper chamber membrane was coated with Matrigel (Millipore) at 37 °C for 6 h before invasion assay. Transwell chambers were kept at 37 °C for 15 h to allow cell invasion and migration. Following staining with 1% crystal violet (Sigma-Aldrich), cell images were obtained using a light microscope.

### Data analysis

Experiments were performed in 3 independent replicates and mean values were used to express all data. Differences were explored between 2 types of tissues (non-tumor vs. NSCLC) using paired t test. ANOVA (one-way) in combination with Tukey test were used to compare more than 2 groups. Linear regression was used to explore correlations. *p* < 0.05 was considered statistically significant.

## Results

### MAGI2-AS3 and RECK were both downregulated and they were correlated in NSCLC

**Fig. 1:**
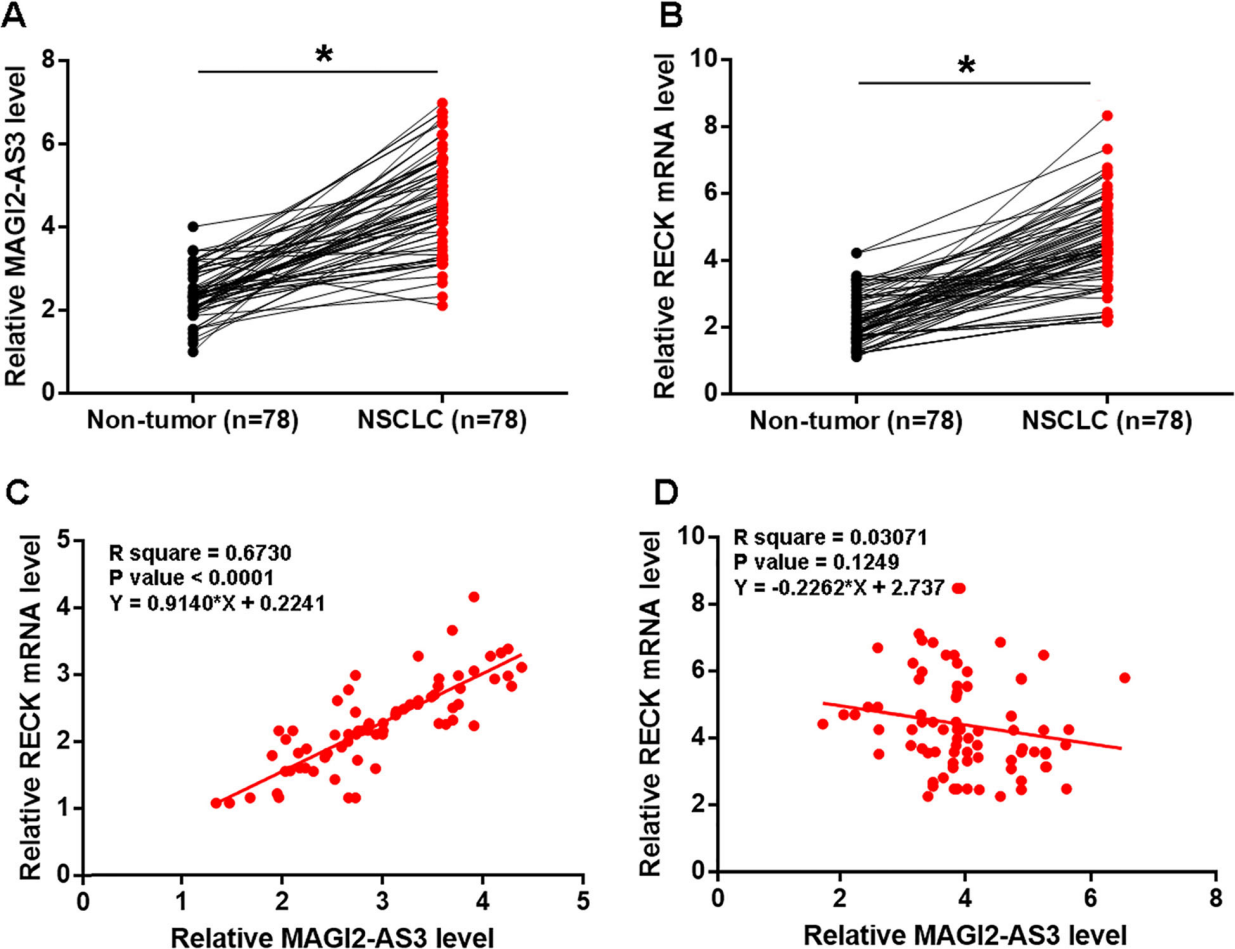
MAGI2-AS3 and RECK were both downregulated and they were correlated in NSCLC. Expression levels of MAGI2-AS3 (**a**) and RECK mRNA (**b**) in two types of tissues (non-tumor and NCSLC) were measured by qPCR. Expression data were compared between two types of tissues by paired t test. Correlations between MAGI2-AS3 and RECK mRNA in NSCLC tissues (**c**) and non-tumor tissues (**d**) were analyzed by linear regression. qPCR was performed 3 times and mean values were presented, *, *p* < 0.05

Expression levels of MAGI2-AS3 and RECK mRNA in two types of tissues (non-tumor and NCSLC) were measured by qPCR. Expression data were compared between two types of tissues by paired t test. It can be observed that expression levels of MAGI2-AS3 (Fig. 1a) and RECK mRNA (Fig. 1b) were significantly lower in NSCLC tissues than those in non-tumor tissues (*p* < 0.05). Correlations between MAGI2-AS3 and RECK mRNA were analyzed by linear regression. It can be observed that, in NSCLC tissues, MAGI2-AS3 and RECK mRNA were significantly and positively correlated (Fig. 1c). However, in non-tumor tissues, MAGI2-AS3 and RECK mRNA were not significantly correlated (Fig. 1d).

### MAGI2-AS3 overexpression led to upregulated RECK in H1993 cells

**Fig. 2:**
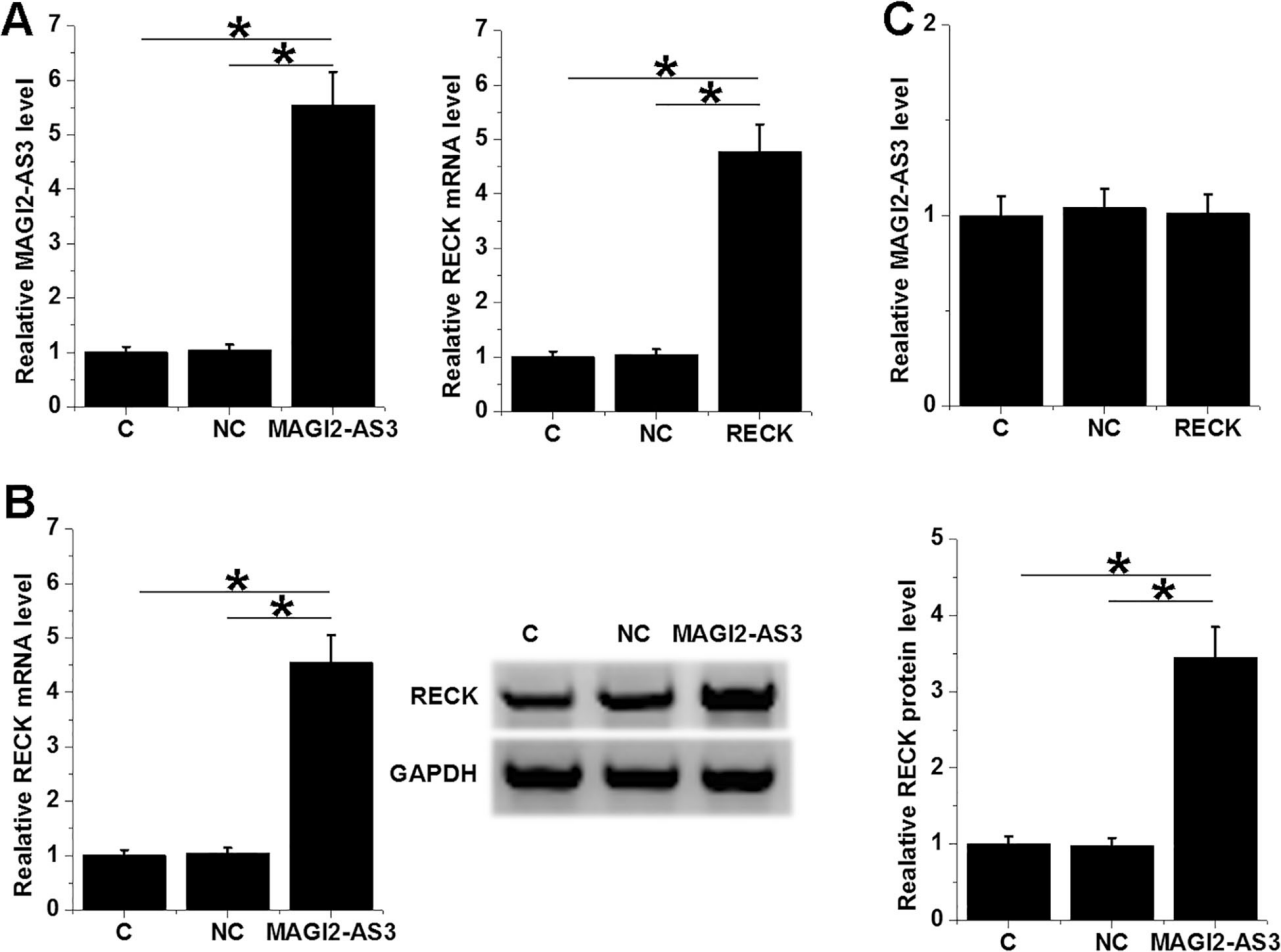
MAGI2-AS3 overexpression led to upregulated RECK in H1993 cells. To further investigate the interaction between MAGI2-AS3 and RECK, MAGI2-AS3 and RECK expression vectors were transfected into H1993 cells. At 24 h post-transfections, overexpression of MAGI2-AS3 and RECK was confirmed by qPCR (**a**). The effects of MAGI2-AS3 overexpression on RECK expression were analyzed by qPCR and western blot (**b**). The effects of RECK overexpression on MAGI2-AS3 expression were analyzed by qPCR (**c**). Western blot and qPCR were performed 3 times and mean ± SD values were presented. *, *p* < 0.05

To further investigate the interaction between MAGI2-AS3 and RECK, MAGI2-AS3 and RECK expression vectors were transfected into H1993 cells. At 24 h post-transfections, expression levels of MAGI2-AS3 and RECK were significantly increased comparing to NC and C groups (Fig. 2a, *p* < 0.05). In addition, MAGI2-AS3 overexpression led to upregulated RECK at both mRNA and protein levels (Fig. 2b, *p* < 0.05). In contrast, MAGI2-AS3 expression was not significantly affected by RECK overexpression (Fig. 2c).

### MAGI2-AS3 may sponge miR-25 to upregulate RECK

**Fig. 3:**
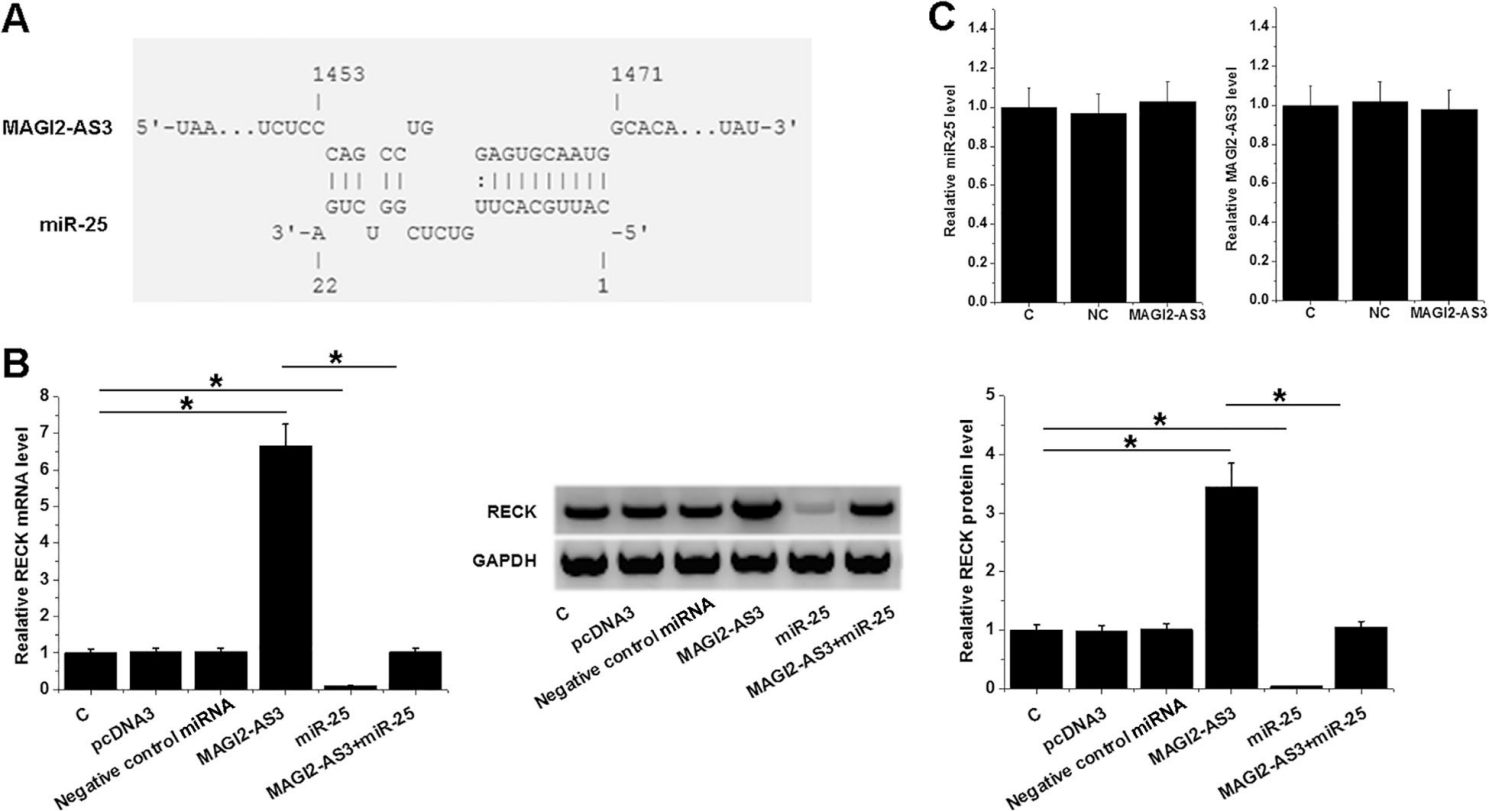
MAGI2-AS3 may sponge miR-25 to upregulate RECK. Bioinformatics analysis showed that MAGI2-AS3 may bind miR-25 (**a**). The interaction between MAGI2-AS3 and miR-25 was explored by performing overexpression and qPCR experiments (**b**). The effects of MAGI2-AS3 and miR-25 overexpression on RECK expression were analyzed by western blot and qPCR (**c**). Western blot and qPCR were performed 3 times and mean ± SD values were presented, *, *p* < 0.05

Bioinformatics analysis showed that MAGI2-AS3 may bind miR-25 (Fig. 3a). Comparing to NC and C groups, MAGI2-AS3 overexpression failed to miR-25 expression, and miR-25 overexpression also failed to affect MAGI2-AS3 expression (Fig. 3b). Moreover, comparing to two controls, miR-25 overexpression led to downregulated RECK and attenuated the effects of MAGI2-AS3 overexpression (Fig. 3c, *p* < 0.05).

### MAGI2-AS3 inhibits NSCLC cell invasion and migration

**Fig. 4:**
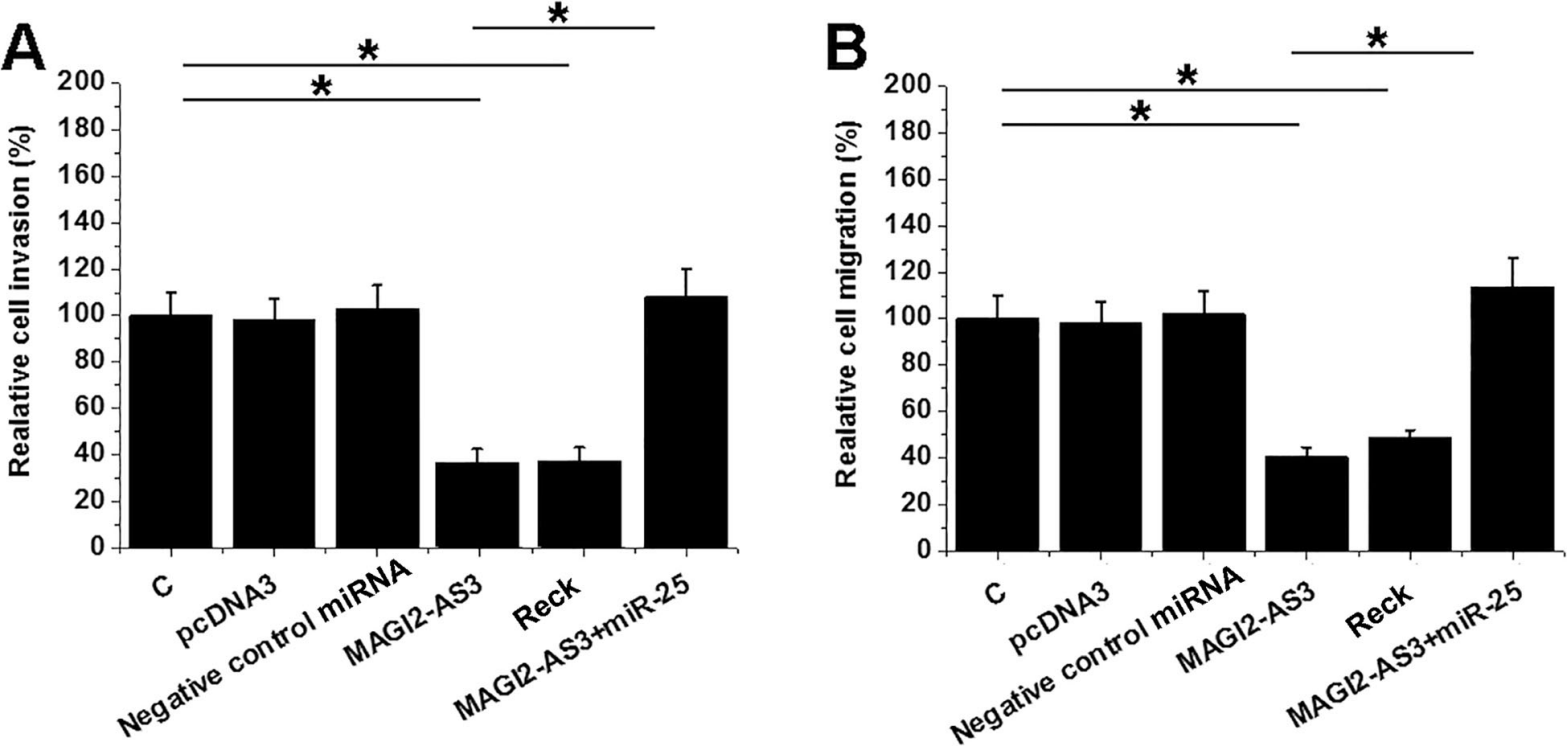
MAGI2-AS3 promotes NSCLC cell invasion and migration. The effects of MAGI2-AS3, miR-25 and RECK overexpression on NSCLC cell invasion (**a**) and migration (**b**) were analyzed by Transwell invasion and migration assays. Experiments were performed 3 times and mean values were presented, *, *p* < 0.05

Cell invasion and migration analysis showed that, comparing to NC and C two groups, NSCLC cell invasion (Fig. 4a) and migration (Fig. 4b) rates were significantly decreased after MAGI2-AS3 and RECK overexpression (*p* < 0.05). MiR-25 reduced the effects of MAGI2-AS3 overexpression (*p* < 0.05).

## Discussion

In this study MAGI2-AS3 was characterized as a tumor suppressor in NSCLC. Moreover, we also found that MAGI2-AS3 may be a sponge of miR-25, and the interaction between MAGI2-AS3 and miR-25 led to upregulated RECK, which is a target of miR-25.

All previous studied on MAGI2-AS3 revealed its tumor suppressive roles in different types of cancers, such as breast cancer, bladder cancer and hepatocellular carcinoma [12–15]. However, MAGI2-AS3 may interact with different downstream targets to regulate different cell behaviors in different types of cancers [13–15]. In breast cancer, MAGI2-AS3 is an inhibitor of cancer cell growth and its roles in this process are mediated by its interactions with Fas/FasL [13]. In bladder cancer, MAGI2-AS3 mainly interacts with CCDC19 to inhibit the migration, proliferation and invasion of cancer cells [14]. In hepatocellular carcinoma, MAGI2-AS3 targets miR-374b-5p/SMG1 to inhibit proliferation and migration of HCC cells [15]. In this study we observed the downregulated expression of MAGI2-AS3 in NSCLC and its inhibitory effects on cancer cell migration and invasion. Our study proved MAGI2-AS3 as a tumor suppressor in NSCLC.

RECK has been characterized as a tumor suppressor in different types of cancers including NSCLC [16, 17]. Some miRNAs, such as miR-96 and miR-92b targets RECK to increase the invasiveness of NSCLC cells [16, 17]. It has been reported that miR-25 can also target RECK in gastric cancer to promote cell motility and growth [11]. In this study we also showed that miR-25 overexpression led to downregulated RECK expression at both mRNA and protein levels. Therefore, miR-25 may also target RECK in NSCLC. However, the upstream regulators of miR-25 in cancer biology have not been well studied. In this study we found that MAGI2-AS3 may sponge miR-25. This conclusion is made due to the observation that MAGI2-AS3 overexpression did not affect miR-25 expression but upregulated its downstream target RECK. However, more studies are needed to further confirm this conclusion.

## Conclusion

In conclusion, MAGI2-AS3 is an tumor suppressor in NSCLC and MAGI2-AS3 may sponge miR-25 to upregulate RECK, thereby promoting NSCLC invasion and migration.

## Data Availability

The analyzed data sets generated during the study are available from the corresponding author on reasonable request.

## Abbreviation

NSCLC: Non-small cell lung cancer
